# Long-term results of percutaneous coronary intervention in no-touch vein grafts are significantly better than in conventional vein grafts

**DOI:** 10.1177/02676591241230012

**Published:** 2024-01-22

**Authors:** Gabriele Ferrari, Håkan Geijer, Yang Cao, Ulf Graf, Leif Bojö, Roland Carlsson, Domingos Souza, Ninos Samano

**Affiliations:** 1 University Health Care Research Centre, Faculty of Medicine and Health, 6233 Örebro University, Örebro, Sweden; 2 Department of Cardiology and Cardiothoracic Surgery, Blekinge Hospital, Karlskrona, Sweden; 3 Department of Radiology, Faculty of Medicine and Health, 6233 Örebro University, Örebro, Sweden; 4 Clinical Epidemiology and Biostatistics, School of Medical Sciences, Faculty of Medicine and Health, 6233 Örebro University, Örebro, Sweden; 5 School of Health and Welfare, 3694 Halmstad University, Halmstad, Sweden; 6 Clinical Physiology Division, Regional Hospital of Karlstad, Karlstad, Sweden; 7 Svensk PCI AB, Regional Hospital of Karlstad, Karlstad, Sweden

**Keywords:** coronary artery bypass graft, major adverse cardiac events, no-touch, percutaneous coronary intervention, saphenous vein, stent

## Abstract

**Introduction:**

Conventional vein grafts have a high risk of thrombosis and early atherosclerosis. Percutaneous coronary intervention (PCI) in conventional vein grafts is associated with a higher incidence of late adverse cardiac events. The aim of this study was to evaluate the long-term results after PCI in saphenous vein grafts (SVG) harvested with the no-touch technique compared to the conventional technique.

**Methods:**

This was a single-center, retrospective, cohort study, based on data from the Swedeheart register. The inclusion criterion was individuals who underwent CABG using different vein graft techniques between January 1992 and July 2020, and who required a PCI in SVGs between January 2006 and July 2020. The primary end point was long-term in-stent restenosis. The secondary endpoints were long-term major adverse cardiac events (MACE) and 1-year re-hospitalization rates. The associations between the graft types and the endpoints were evaluated using the Fine and Gray competing-risk regression analysis.

**Results:**

The study included 346 individuals (67 no-touch, 279 conventional). The mean clinical follow-up time was 6.4 years with a standard deviation of 3.7 years. The long-term in-stent restenosis rate for the no-touch grafts was 3.2% compared to 18.7% for the conventional grafts (*p* < .01), with a subdistribution hazard ratio (SHR) of 0.16 (*p* = .010). The long-term MACE rate was 27.0% in the no-touch group and 48.3% in the conventional group (*p* < .01) with a SHR of 0.53 (*p* = .017). The short-term results were similar in both groups.

**Conclusions:**

Percutaneous coronary intervention in a no-touch vein graft was associated with statistically significantly fewer in-stent restenoses and MACE at long-term follow-up compared to a conventional SVG.

## Introduction

Saphenous vein grafts (SVGs) are the most commonly used grafts, alongside left internal thoracic artery grafts (LITA) during a coronary artery bypass grafting (CABG) operation. The vein grafts are susceptible to early stenosis and have limited patency due to remodelling, progressive intimal hyperplasia and atherosclerosis.^1–3^; with a subsequent need for repeated coronary angiography interventions. Percutaneous coronary intervention (PCI) is a safe and established procedure, in particular regarding the native coronaries.

Percutaneous coronary intervention of SVGs, on the other hand, results in a complex procedure and its use has been debated in reports,^4^ being associated with controversial results and high rates of major adverse cardiac events (MACE) in both the short and long term. The challenges related to a PCI in SVG are related to the frequent presence of multiple stenosis in the graft, the extension in length of the stenosis, and the complexity of performing a PCI at the anastomosis site.

In a recent systematic review and meta-analysis,^4^ we reported significantly higher rates of events after PCI in a SVG, compared with the stenting of native coronary vessels. The pooled MACE value at 2.7 years after stenting of vein grafts was 35.3%, compared to 15% in the native vessel group. The pooled in-stent restenosis at long term follow-up was 9.4%, compared to 3.2% in the control group.^4^

No results have yet been described concerning PCI in a no-touch (NT) vein graft. The NT technique^5^ results in a more careful harvesting and less endothelium damage^6,7^; this implies less intimal hyperplasia and consequently less atherosclerosis at follow-up.^8–10^

The aim of this study is to evaluate the long-term results after PCI in SVGs harvested with the NT technique and to compare these results with PCI in conventional (C) SVGs.

The hypothesis is that the NT SVGs are prone to an easier PCI and subsequently to a reduced rate of restenosis.

## Materials and methods

### Data collection

This is a single-center, retrospective, cohort study. The study complied with the STROBE (STrengthening the Reporting of OBservational studies in Epidemiology) statement and the STROBE checklist was used.^11^

The study inclusion criterion was all patients receiving a PCI in a SVG (stenosed or occluded) between 1 January, 2006 and 30 June, 2020. The cohort included all patients who had undergone CABG operation at our Cardiothoracic Unit between January 1992 and May 2020. The Regional Ethical Review Board (Regionala etikprövningsnämnden i Uppsala) approved the study (Dnr 2015/242 and Dnr 2020/04168).

The SVG was harvested using either the NT or the C technique.^12^ We used a totally open technique for both the harvesting methods, no bridge techniques or endoscopical techniques were used (Supplemental Figure 1). The NT grafts were harvested with a 2–5 mm wide fat pedicle. The grafts were not distended with saline solution. The anastomosis leak test was performed with cardioplegia and with the actuarial extracorporeal circulation pressure; no manual or external pressure was used.

The C grafts were harvested by stripping them from their surrounding tissues. They were then dilated with saline solution to overcome the spasm due to the harvesting. The anastomosis leak test was performed using saline solution or cardioplegia, usually with manual pressure.

The type of technique used was chosen by the surgeon at the time of operation based on individual assessment, and therefore without randomization. The standard operation in our center has been to anastomise the LIMA to the LAD, and the vein graft (harvested using either the NT or C technique) to the other target vessels; no standard double mammary or radial artery was used. Two cardiac surgery specialists reviewed all the operating records to assess the exact partition in the two groups. The PCIs were carried out in two different cardiology units. The PCI was performed depending on clinical need, such as acute myocardial infarction (MI) or recurrent stable angina not responding to medical therapy; it was also clinically driven in cases of re-angiography. No planned angiographic follow-up was performed.

The only exclusion criterion was if PCI was performed during the first month after CABG since this was determined to be a technical issue of the surgery, and therefore not related to the vein graft type or to the harvesting technique. If the patient had undergone more than one PCI procedure, the first procedure after CABG was analyzed.

The data extraction regarding the operative (CABG), procedural (PCI), and periprocedural data was performed with the help of an intervention-related quality register (the Swedish cardiological and cardiosurgical intervention register Swedeheart).^13^ All coronary angiography images were double-checked by an expert radiologist to verify the veracity of the PCI primary clinical report regarding the criteria of vein graft stenosis and the success, or not, of the stenting procedure. Follow-up was conducted up until 31 May, 2021 by reviewing the clinical software of the two hospitals involved and accessing the Swedish Cause of Death Register^14^ for the assessment of cause of death.

The study involved the center that developed the NT technique and has been using it since 1990 for harvesting of the saphenous vein. The study was performed according to the Declaration of Helsinki.

### Statistical methods

The primary endpoint was defined as long-term in-stent restenosis.

The secondary endpoints were the long-term MACE (defined as cardiac death, myocardial infarction, or target vessel revascularization) rate and re-hospitalization rate at 1 year; both these parameters are therefore strictly related to the graft durability, the patients’ quality of life, and the economic impact on the healthcare system.^15^

Other outcomes were long-term results such as mortality, re-angina, and re-angiography rates; short-term results such as 30-days MACE, 30-days re-angina, 1-year MACE, 1-year in-stent restenosis, 1-year re-angina, and 1-year re-angiography rate; and periprocedural success rate.

Descriptive statistics were calculated as means ± standard deviations (SD) for normally distributed variables, as median with interquartile range (IQR) for non-normally distributed variables, and as count with percentage for categorical variables.

Chi-squared test was used to compare categorical proportions between the groups (NT and C). Fisher’s exact test was used where the expected count was lower than 5. Unpaired *t*-test was performed to compare continuous variables and Mann-Whitney U-test was used for non-normally distributed data.

The survival analysis for both primary and secondary outcomes was conducted using the Fine and Gray competing-risks regression analysis, with death accounted for as a competing risk. To illustrate the cumulative incidence rates of the interested outcomes across different graft groups, cumulative incidence curves were employed.

The associations between the graft types and the primary and secondary outcomes were evaluated using subdistribution hazard ratios (SHRs) with 95% confidence interval (CIs) in the competing-risk regression analysis, adjusted for age, sex, diabetes, hypercholesterolemia, hypertension, smoking, the type of stent, the number of stents used, and the years between CABG and PCI. The proportional hazards assumption was tested by using time-dependent covariates in addition to the standard covariates. For covariates that did not meet the assumption, interaction terms between time and the covariates were added into the competing-risk regression models.

All the statistical analyses were performed using SPSS version 27.0 (IBM, Armonk, NY, USA) or Stata 18.0 (StataCorp, College Station, TX, USA), and an SHR with a two-sided *p*-value less than 0.05 was considered as statistically significant.

## Results

**Figure 1.**
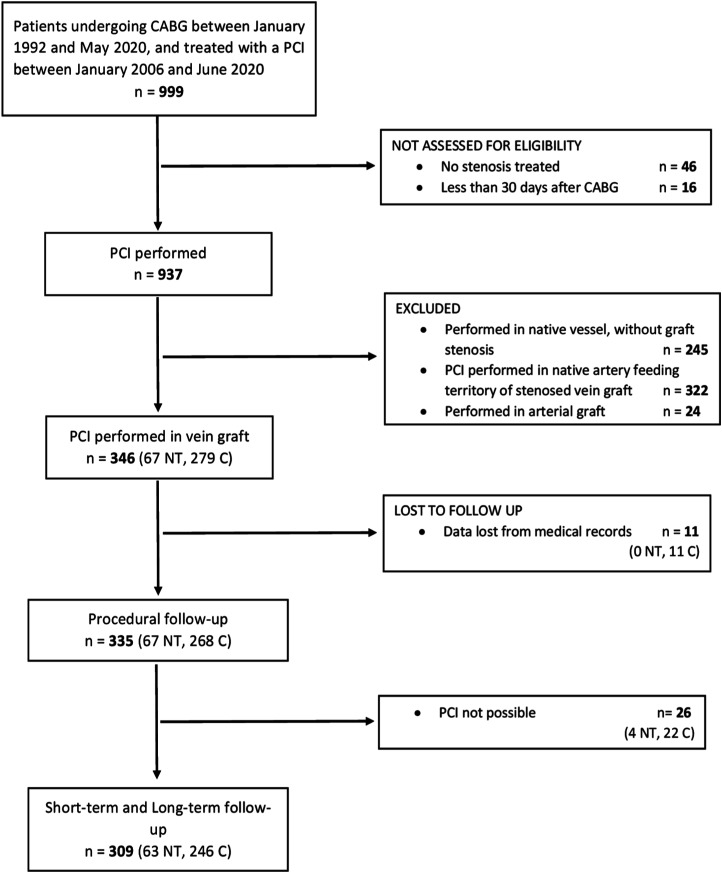
STROBE flowchart of the individuals included in the study. C, conventional graft; CABG, coronary artery bypass grafting; NT, no-touch graft; PCI, percutaneous coronary intervention.

Between 1 January, 2006 and 30 June, 2020, 937 individuals who had previously been treated with CABG were subsequently treated with a PCI (Figure 1). The study included 346 consecutive patients (67 NT, 279 C) treated with a PCI in a stenosed or occluded SVG.

Excluding 11 individuals who were lost at follow-up, a total of 335 were available for the analysis (67 NT, 268 C). In 26 cases (4 NT (7.5%), 22 C (8.2%)) it was impossible to perform a PCI because it was not feasible to overcome the stenosis/occlusion or there was difficulty in dilating it. These 26 cases were included in the analysis of periprocedural mortality and morbidity, but not in the clinical follow-up. The mean clinical follow-up was 6.4 ± 3.7 years (up to 15.1 years) and included 309 patients (63 NT, 246 C).

### Demographic, operative, and procedural characteristics

The demographic, operative and procedural (PCI) characteristics of the population are summarized in Table 1 (and Supplemental Table 1).

**Table 1.** Demographic, operative and procedural characteristics divided by group.

Patient characteristics	No-touch	Conventional	*p*-value
No. of patients	67	268	
Mean age at CABG, years ± SD	65.7 ± 10.1	63.3 ± 10.8	.107
Years between CABG and PCI, mean ± SD	12.1 ± 6.2	14.2 ± 4.8	.003
Male, *n* (%)	53 (79.1)	219 (81.7)	.625
Hypertension, *n* (%)	61 (91)	232 (86.6)	.322
Hypercholesterolemia, *n* (%)	61 (91)	223 (83.2)	.110
Diabetes mellitus, *n* (%)	19 (28.4)	105 (39.2)	.101
Smoking, *n* (%)	31 (42.3)	136 (50.7)	.388
DAPT at PCI time, *n* (%)	37 (55.2)	182 (67.9)	.051
Creatinine mean ± SD	87.9 ± 26.3	100.1 ± 56.8	.101
Occlusions, *n* (%)	26 (38.8)	146 (54.5)	.022
DES, *n* (%)	53 (79.1)	198 (73.9)	.785^b^
Number of stents, *n*	101	373	.411^b^
Thrombectomy, *n* (%)	7 (10.4)	26 (9.7)	.855
EPD, *n* (%)	2 (3.0)	12 (4.5)	.744^b^
N. peripheral anastomosis *n* (%)			.365^a^
1	0 (0.0)	3 (1.1)	
2	4 (6.0)	16 (6.0)	
3	27 (40.3)	97 (36.2)	
4	22 (32.8)	115 (42.9)	
5	11 (16.4)	36 (23.4)	
6	3 (4.5)	1 (0.4)	
PCI not possible, *n* (%)	4 (6.0)	22 (8.2)	.798^b^

*t*-test was used for continuous variables and chi-squared test for categorical variables. Data are shown as mean ± standard deviation or *n* (%). Ages and times are provided in years. Creatinine is provided in micromole/L.

DAPT: double platelet inhibitor therapy; DES: drug-eluting stent; EPD: embolic protection device; PCI: percutaneous coronary interventions.

^a^Mann-Whitney U test was used.

^b^Fisher’s exact test was used.

The patients were of similar age at the time of CABG (65.7 ± 10.1 years NT, 63.3 ± 10.8 years C; *p* = .107; Table 1); the patients’ other demographic characteristics were also comparable. Most of the patients were male (81.2%). The risk factors were also similarly distributed. Although the NT group contained a higher percentage of patients with hypertension and a smaller number with diabetes, all the comparisons resulted in a statistically non-significant *p*-value except for number of days between CABG and PCI. The PCI procedure in the NT group was performed at a mean of 12.1 ± 6.2 years after the CABG operation, but at a mean of 14.2 ± 4.8 years in the C group (*p* = .003; Table 1). The significantly (*p* = .022) higher presence of graft occlusion at the moment of PCI in the C group is notable.

The operative characteristics regarding the number of peripheral anastomoses were comparable in the two groups (Table1). No statistically significant differences were noted in the coronary target territory for the two types of vein graft (SVG to right coronary artery: *p* = .053; SVG to circumflex coronary artery: *p* = .471; SVG to diagonal coronary artery *p* = .274).

The procedural characteristics (the number of drug-eluting stents (DES), the total number of implanted stents, the number of thrombectomies, and the number of distal embolic protection devices (EPD) used) were also similar (Table 1). The very limited number of EPD used in our centers is worth noting: 14 cases (2 NT, 12 C).

### Clinical follow-up

#### Primary endpoint

The in-stent restenosis rate at clinical follow-up at a mean of 6.4 ± 3.7 years was 3.2% (2 cases) in the NT group and 18.7% (46 cases) in the C group (*p* < .01, Table 2).

**Table 2.** Long-term results at mean follow-up time, 1-year, and 30-days results.

Patient characteristics	No-touch	Conventional	*p*-value*
No. of patients	63	246	
Re-angina (effort angina), *n* (%)	10 (15.9)	74 (30.1)	.026
MACE, *n* (%)	17 (27)	119 (48.4)	.003
In-stent restenosis, *n* (%)	2 (3.2)	46 (18.7)	.001
TVR, *n* (%)	5 (7.9)	59 (24)	.005
STEMI/NSTEMI, *n* (%)	13 (20.6)	79 (32.1)	.090
Cardiac death, *n* (%)	5 (7.9)	47 (19.1)	.038
Total death, *n* (%)	15 (23.8)	97 (39.4)	.027
Re-angiography during first year, *n* (%)	4 (6.3)	41 (16.7)	.044
Re-hospitalization during first year, *n* (%)	8 (12.7)	71 (28.9)	.009
MACE 1-year, *n* (%)	7 (11.1)	41 (16.7)	.333
In-stent restenosis 1-year, *n* (%)	2 (3.2)	12 (4.9)	.743
TVR 1-year, *n* (%)	3 (4.8)	15 (6.1)	1.000
STEMI/NSTEMI 1-year, *n* (%)	4 (6.4)	29 (11.8)	.259
Total death 1-year, *n* (%)	2 (3.2)	4 (1.6)	.606
Unsuccessful PCI (TIMI flow <2), *n* (%)	3 (4.8)	15 (6.1)	1.000
MACE 30-days, *n* (%)	2 (3.2)	15 (6.1)	.539
In-stent restenosis 30-days, *n* (%)	0 (0.0)	1 (0.4)	1.000
TVR 30-days, *n* (%)	0 (0.0)	2 (0.8)	1.000
STEMI/NSTEMI 30-days, *n* (%)	2 (3.2)	11 (4.5)	1.000

MACE, major adverse cardiac events; NSTEMI, non-ST-elevation myocardial infarction; STEMI, ST-elevation myocardial infarction; TVR, target vessel revascularization.

*Fisher’s exact *p*-value. Values are presented as *n* (%).

#### Secondary endpoints

The rate of MACE at long-term follow-up was significantly lower in the NT group (27%), compared to the C group (48.4%); *p* = .003 (Table 2).

At least one re-hospitalization during the first year after PCI was twice as frequent in the C group (12.7% NT, 28.9% C, *p* = .009) (Table 2).

#### Peri-procedural and 30 days results

It was not possible to perform PCI in 4 cases (6%) in the NT group and in 22 cases (8.2%) in the C group; *p* = .54 (Table 1).

The rate of non-success, specified as Thrombolysis in Myocardial Infarction (TIMI) flow <2, was similar in the two groups (4.8% NT, 6.1% C (*p* = 1.000).

The number of cardiac events (MACE, acute myocardial infarction, in-stent restenosis, and target vessel revascularization (TVR),) was also comparable in the two groups for each endpoint (Table 2).

#### Short-term follow-up

The 1-year follow-up results were similar in the two groups, except for the already mentioned re-hospitalization rate (Table 2).

#### Long-term follow-up

The long-term results, at 6.4 ± 3.7 years, are summarized in Table 2. The NT group was affected by fewer cardiac events, with a statistically significant different rate of re-angina (15.9% NT vs 30.1% C; *p* = .026), rate of TVR (7.9% NT vs 24.0% C; *p* = .005), number of cardiac deaths (7.9% NT vs 19.1% C; *p* = .038), and number of total deaths (23.8% NT vs 39.4% C; *p* = .027). The rate of acute MI at long-term follow-up was also numerically lower in the NT group (20.6% NT vs 32.1% C), but the difference was not significant (*p* = .090).

#### Survival analysis

**Figure 2.**
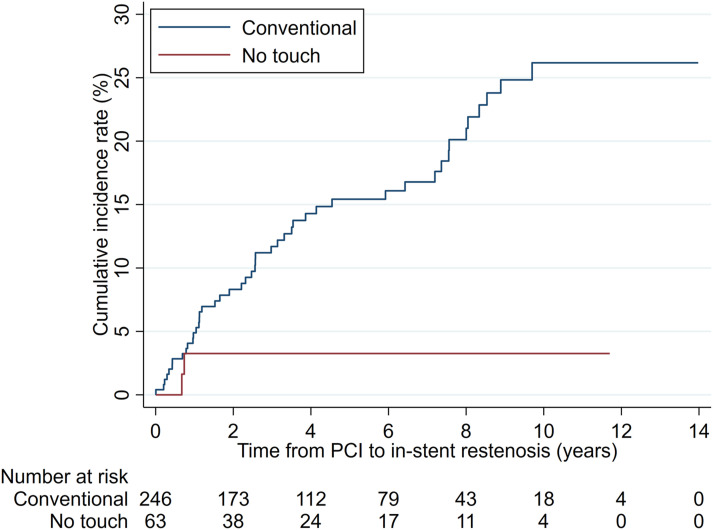
Cumulative incidence rates of in-stent restenosis by graft groups.

The competing-risks regression analysis adjusted for covariables confirmed a statistically significant difference between the groups (SHR = 0.16, 95% CI: 0.04–0.65; *p* = .010) with a cumulative incidence rate of in-stent restenosis at 8 years of 3.1% (NT) and 20.0% (C), respectively (Figure 2).

**Figure 3.**
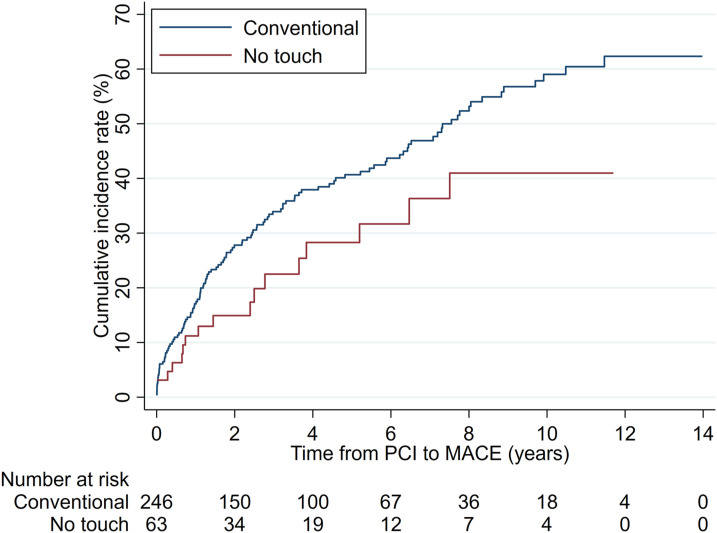
Cumulative incidence rates of MACE (major adverse cardiac events) by graft groups.

The cumulative incidence rate of MACE at 12 years was 41.1% (NT) and 63.2% (C), respectively (Figure 3). The competing-risks regression analysis adjusted for covariables confirmed a statistically significant difference (SHR = 0.53, 95% CI: 0.31–0.89; *p* = .017); Figure 3.

The time to event analyses for the remaining secondary outcomes are shown in Supplemental Figures 2 and 3.

**Figure 4.**
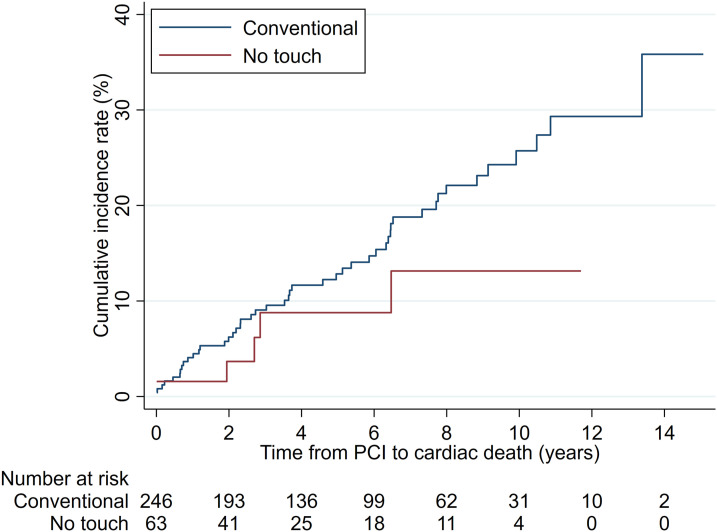
Cumulative incidence rates of cardiac death by graft groups.

The cumulative incidence rate for cardiac death at 12 years was 13.1% (NT) and 35.9% (C), respectively (Figure 4); However, the competing-risks regression analysis adjusted for covariables did not reveal a statistically significant difference (SHR = 0.46, 95% CI: 0.19 – 1.13; *p* = .092).

Moreover, the Cox regression adjusted for covariables were performed for other secondary outcomes (Supplemental Figures 4 and 5), confirming the results of the univariate analysis.

## Discussion

This is the first study investigating the results of percutaneous intervention of a NT SVG compared to C SVG.

The major finding of this study was that patients with a NT SVG had significantly lower risk for in-stent restenosis, being as low as one-sixth (SHR = 0.16) at long-term follow-up (6.4 ± 3.7 years), according to the competing-risks regression analysis adjusted for covariables.

The rate of re-angiography at 1 year (*p* = .044) and the rate of re-hospitalization at 1 year (*p* = .009) also showed statistically significant differences.

C SVGs have a high risk of intimal hyperplasia and thrombosis, which lead to graft occlusion rates after 10 years of up to 50–60% in most clinical records.^16–18^ PCI in SVGs is under continuous evolution and currently corresponds to approximately 6% of the percutaneous coronary procedures performed in the US.^19^ Patients who needed a PCI in their SVGs have a higher risk of MACE in comparison with those experiencing PCI in native coronary arteries. On the other hand, in many cases a PCI in the native vessel is not feasible^20^; a PCI in SVG can therefore be the only solution, despite the higher risk for adverse cardiac events.^21,22^

A recent meta-analysis^4^ confirmed that PCI in a stenosed SVG is associated with high rates of procedural failure, short- and long-term cardiac events, and restenosis, despite developments in stent types and in the PCI methods. The in-stent restenosis rate was found to be 9.4% (95% CI: 4.2–14.7%) and the MACE rate 35.3% (95% CI: 27–43.7%) at a mean time of 2.7 ± 1.0 years.

In our study, the primary endpoint results (long-term in-stent restenosis) of 3.2% for the NT grafts match the lowest rates presented in the literature (for instance 2.7% in a study by Redfors et al.^23^) and were lower than the pooled value presented in the literature of 9.4% (95% CI: 4.2–14.7%).^4^

A recent study by Nusca et al.^24^ highlights how in-stent restenosis negatively impacts the patients’ long-term clinical outcomes.

The secondary endpoint results (long-term MACE) for the NT grafts (27%) were better than the pooled result presented in the international literature (35.3%)^4^ and showed a statistically significant difference compared with the C grafts in the same cohort (*p* ≤ .01), with a hazard ratio of 0.48.

The MACE rate at long-term follow-up for the NT group was comparable to the results of PCI in native vessels of the large post-CABG cohort study published by Brilakis et al.,^21^ where the 5-years MACE rate was 37.9%.

The remaining long-term results were better than the pooled values presented in the international literature^4^ for the NT, for each of the outcomes. The comparison between the two groups showed better outcomes for NT, and with a statistically significant difference regarding TVR (*p* ≤ .01). The short-term outcomes were similar in the NT and C SVGs in the cohort, with rates comparable with the literature.^4^ The sole short-term result showing a statistically significant difference between the grafts was the 1-year re-hospitalization rate, which was due to very high rates in the C SVG group (28.9%).

Our results can partially be explained by the fact that the time between CABG and PCI was shorter in the NT group (12.1 vs 14.2 years; *p* = .003).

Seventy-five percent of the stents used were DES; this is equivalent to other reports^4^ and is comparable with the international literature.

The NT SVGs were treated with more DES (79.1% NT vs 73.9% C), but the difference was not statistically significant; *p* = .785. The need for thrombectomy and the use of distal embolic protection were also similar in the two groups. Total occlusions were treated in the same way in the two groups, with standard PCI if possible, and very rare use of thrombectomy.

Several studies^6,8–10,25^ have shown the superiority of NT SVG compared to C SVG on follow-up at a mean time of 16 years. The background for the study hypothesis is the decreased vascular smooth muscle cell activation when the vein graft is harvested with a NT technique, as observed in the PATENT SVG trial.^10^ The better results in PCI in NT SVGs, especially in terms of in-stent restenosis, may play an important role in reduced long-term MACE and re-hospitalization rates.

During the reviewing process of the coronary angiographies, our group noted that the stenoses in NT grafts were mostly localized and single and not as diffuse as in C SVGs (Supplemental Figure 6). We have also observed in postmortem autopsies that the remodelling and atherosclerosis processes in NT SVGs are very different in comparison to the C grafts. These findings may play a fundamental role for the better long-term results of PCI in NT SVGs.

The main limitation of this study is the limited number of NT vein grafts treated with PCI, compared to the number of C grafts. A limitation is also the non-randomized study design.

Another limitation is the temporal bias. In the early 1990s, the C technique was used more frequently than the NT. This is reflected in our study with more slightly older grafts being carried out using the C technique compared to the NT technique. It also implies benefits for the NT grafts having gained the advantages of slightly more modern PCI techniques.

Our future intention is to assess approximately one thousand patients who have undergone a coronary angiography due to angina after their CABG operation. This may reveal reasons for the limited number of NT SVGs being treated with PCI.

### Conclusions

The results regarding PCI in a NT SVG are encouraging, with statistically significantly fewer in-stent restenoses and MACE at long-term follow-up compared with PCI in a C SVG.

The results suggest an important reduction of cardiovascular events after treatment. This may lead to benefits at both individual level (fewer episodes of re-angina and myocardial infarction, and fewer coronary interventions), as well as at community level (fewer re-hospitalizations).

## Supplemental Material


Supplemental Material - Long-term results of percutaneous coronary intervention in no-touch vein grafts are significantly better than in conventional vein grafts
Supplemental Material for Long-term results of percutaneous coronary intervention in no-touch vein grafts are significantly better than in conventional vein grafts by Gabriele Ferrari, Håkan Geijer, Yang Cao, Ulf Graf, Leif Bojö, Roland Carlsson, Domingos Souza and Ninos Samano in Journal of Perfusion.

## Appendix

### Abbreviations

ACS: Acute coronary syndromes
C: Conventional
CABG: Coronary artery bypass grafting
CI: Confidence interval
DAPT: Dual antiplatelet therapy
DES: Drug-eluting stent
EPD: Embolic protection device
IQR: Interquartile range
LITA: Left internal thoracic artery
MACE: Major adverse cardiac events
MI: Myocardial infarction
NT: No-touch
PCI: Percutaneous coronary intervention
SD: Standard deviation
SVG: Saphenous vein graft
TIMI: Thrombolysis in myocardial infarction
TVR: Target vessel revascularization.
