# Supplementary material for: Long-term results of percutaneous coronary intervention in no-touch vein grafts are significantly better than in conventional vein grafts
Source: Perfusion. 2024 Jan 22;40(1):211–20. doi: 10.1177/02676591241230012 (PMC11715067; doi:10.1177/02676591241230012)
Supplement: Supplemental Material - Long-term results of percutaneous coronary intervention in no-touch vein grafts are significantly better than in conventional vein grafts [file sj-pdf-1-prf-10.1177_02676591241230012.pdf]

Supplementary Fig. 1: conventional and no-touch harvesting technique

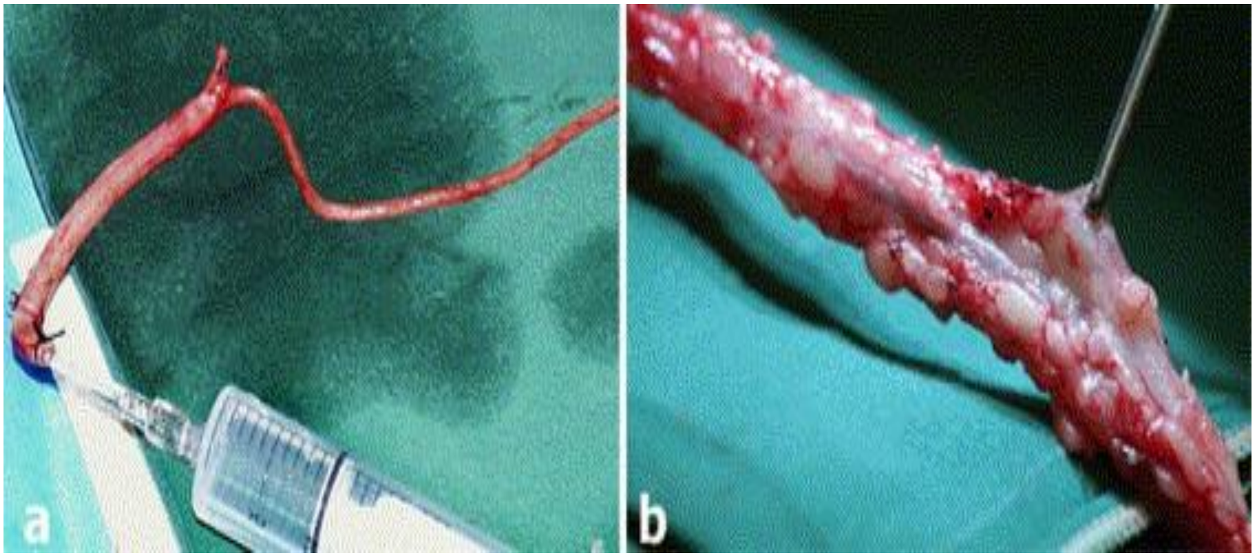

Supplementary Table 1: Demographic characteristics of the patients who received a successful PCI

**Patient characteristics**	**No-touch**	**Conventional**	**p-value***
No. of patients	63	246	
Mean age at CABG, years ±SD	66.2 ± 9.7	63.4 ± 10.8	0.067
Days between CABG and PCI, mean ± SD	4551 ± 2211	5228 ± 1721	0.009
Male, n (%)	50 (79.4)	197 (80.1)	0.899
Hypertension, n (%)	57 (90.5)	212 (86.2)	0.365
Hypercholesterolemia, n (%)	57 (90.5)	203 (82.5)	0.123
Diabetes mellitus, n (%)	18 (28.6)	100 (40.7)	0.078
Double platelet inhibitor therapy, n (%)	34 (54)	169 (68.7)	0.028
Creatinine, mean ± SD	88.4 ± 26.6	100.9 ± 59.0	0.119

*t-test was used for continuous variables and Chi-squared test was used for categorical variables.

Values are presented as mean ± standard deviation or n (%). Ages and times are given in years or days. Creatinine is given in micromole/L. CABG= coronary artery bypass grafting; PCI= percutaneous coronary intervention.

Supplementary Fig. 2: Kaplan-Meier curves of time to MI

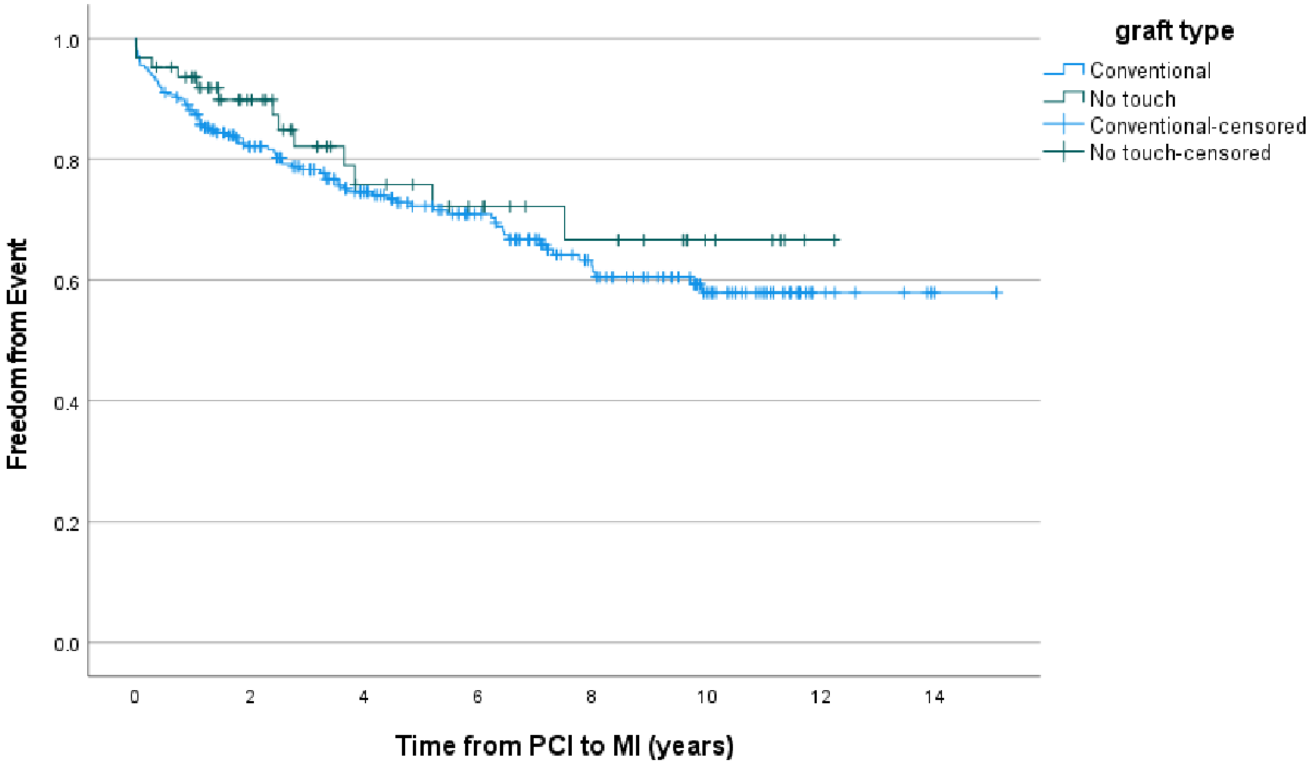

**Years**	**0**	**2**	**4**	**6**	**8**	**10**	**12**	**14**
**No at Risk:**								
**NT:**	**246**	**177**	**134**	**102**	**67**	**40**	**8**	**1**
**C:**	**63**	**40**	**23**	**17**	**12**	**6**	**1**	

Log-rank= 0.344.

MI= myocardial infarction.

Supplementary Fig. 3: Kaplan-Meier curves of time to TVR

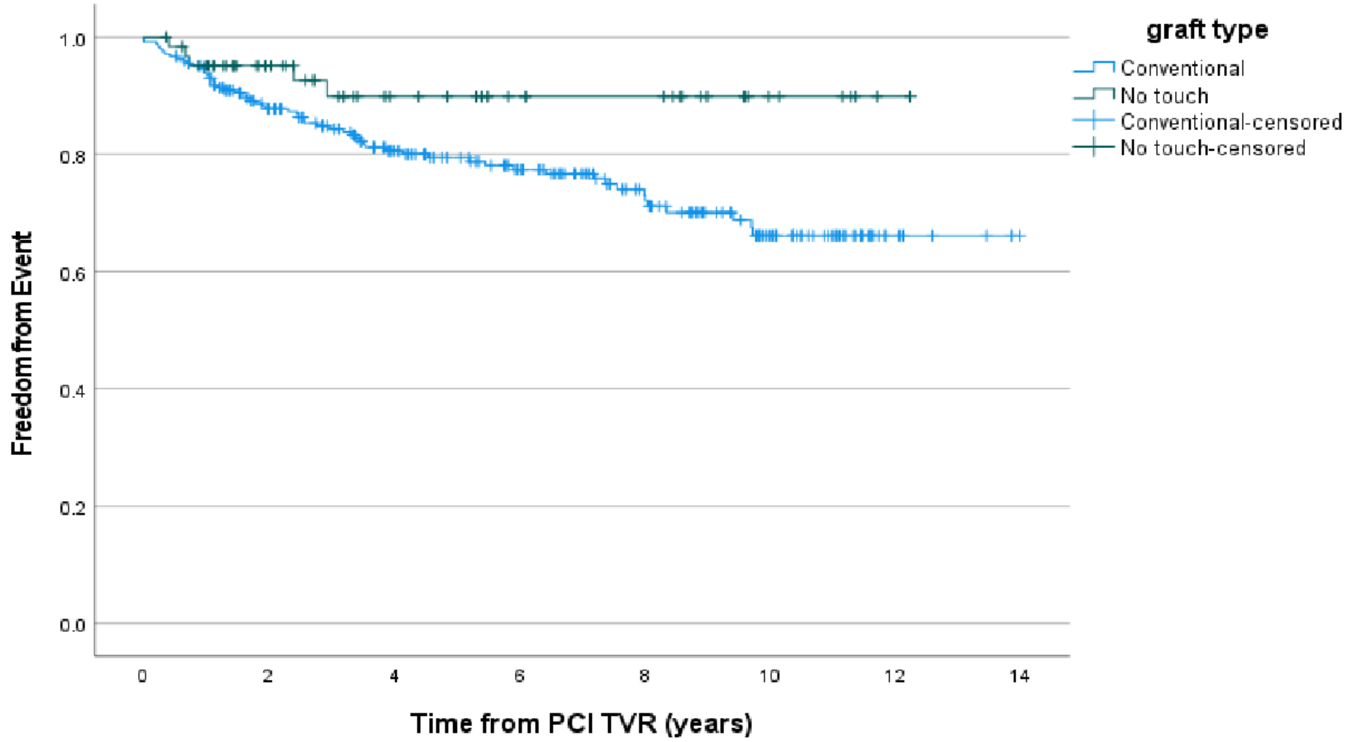

**Years**	**0**	**2**	**4**	**6**	**8**	**10**	**12**
**No at Risk:**							
**NT:**	**246**	**189**	**142**	**110**	**76**	**42**	
**7**							
**C:**	**63**	**42**	**25**	**18**	**16**	**6**	**1**

Log-rank= 0.037.

TVR= target vessel revascularization.

Supplementary Fig. 4: Cox regression analysis of time to re-angina (adjusted)

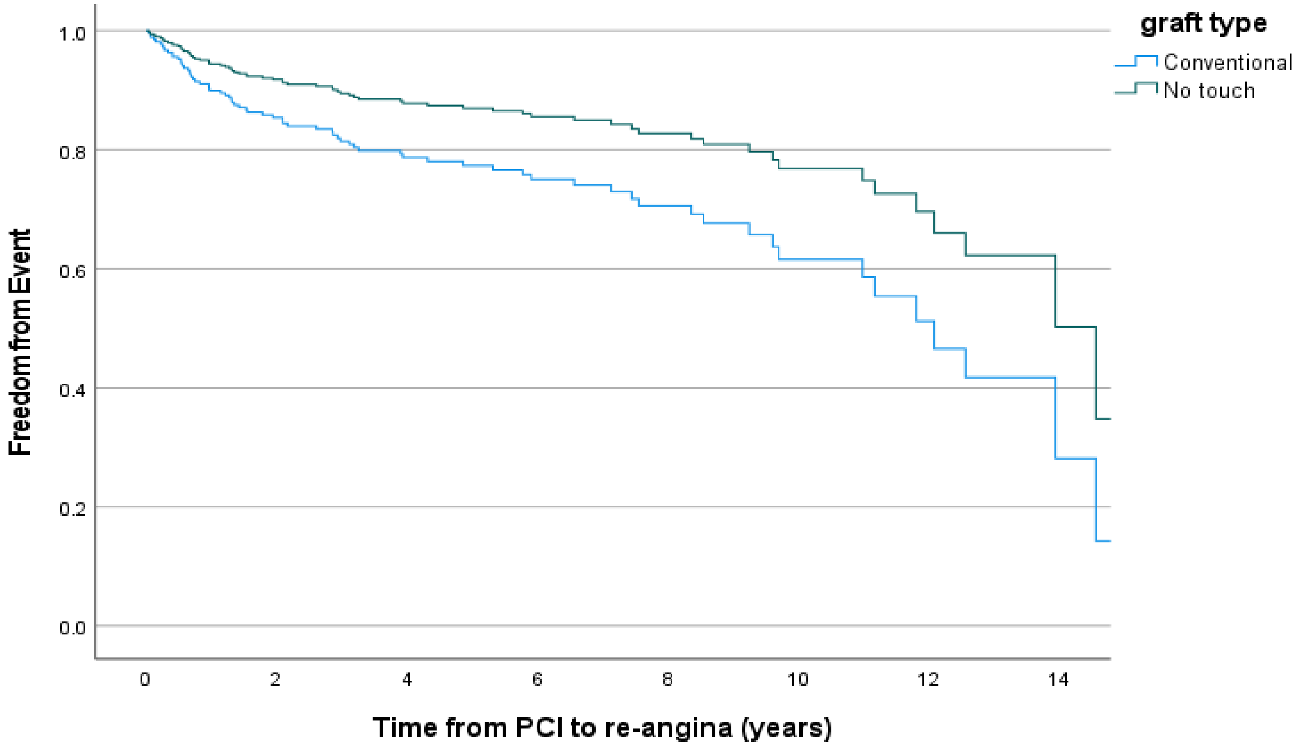

Supplementary Fig.5: Cox regression analysis of time to MI (adjusted)

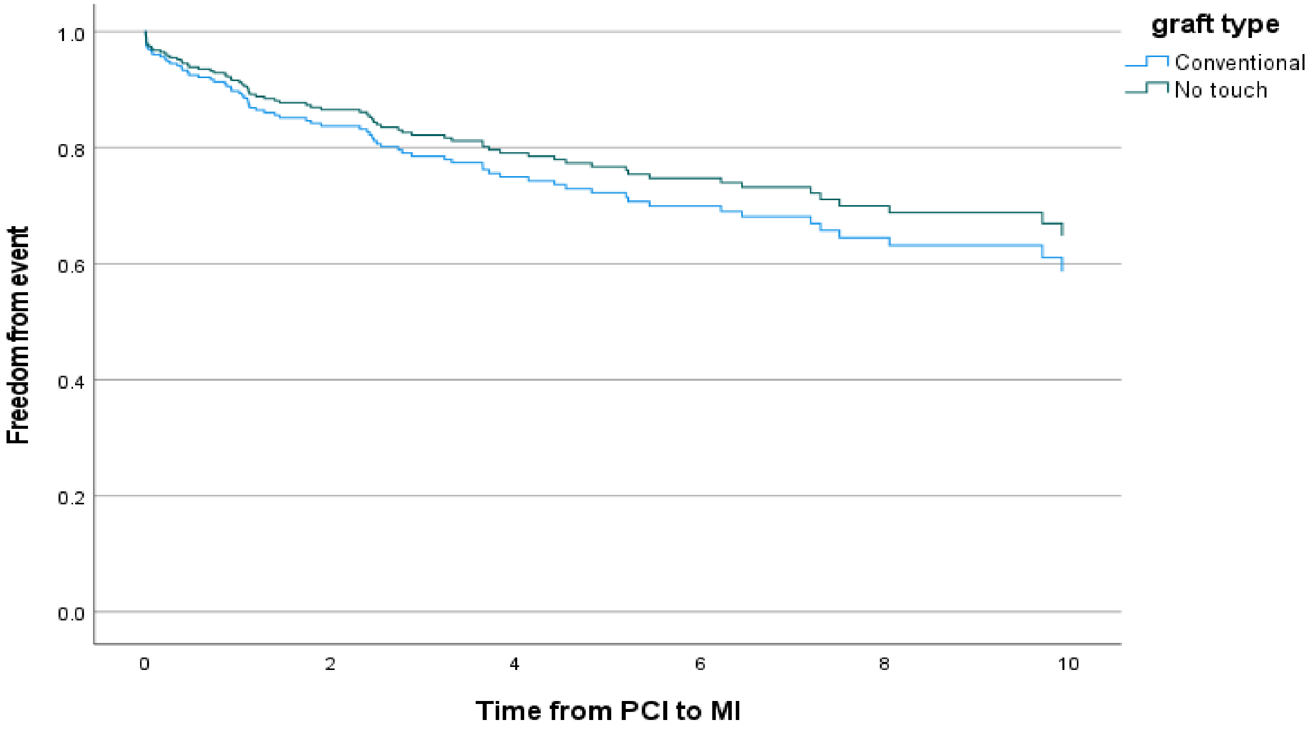

MI= myocardial infarction.

Supplementary Fig. 6: A stenosed no-touch vein graft before (A) and after (B) percutaneous coronary intervention. Arrow shows the stenosis.

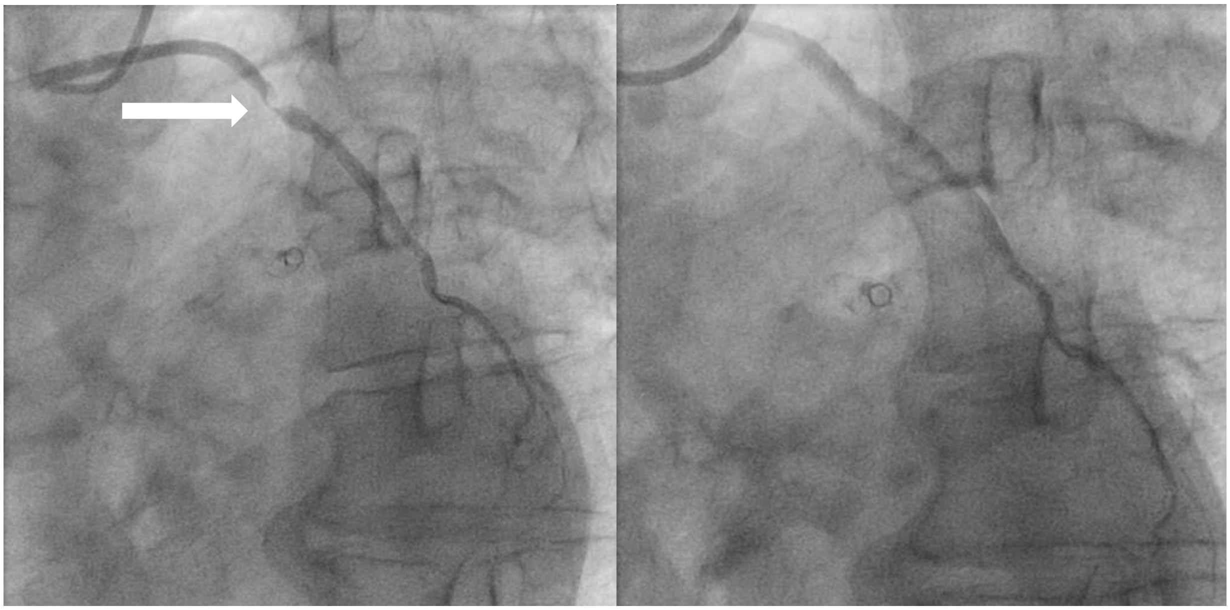

A **B**
